# Health Insurance Coverage Better Protects Blacks than Whites against Incident Chronic Disease

**DOI:** 10.3390/healthcare7010040

**Published:** 2019-03-10

**Authors:** Shervin Assari, Hamid Helmi, Mohsen Bazargan

**Affiliations:** 1 Department of Family Medicine, Charles R. Drew University of Medicine and Sciences, Los Angeles, CA 90059, USA; mohsenbazargan@cdrewu.edu; 2 Center for Research on Ethnicity, Culture and Health, School of Public Health, University of Michigan, Ann Arbor, MI 48109, USA; 3 School of Medicine, Wayne State University, Detroit, MI 48202, USA; hhelmi@umich.edu; 4 Department of Family Medicine, University of California Los Angeles, Los Angeles, CA 90095, USA

**Keywords:** race, ethnicity, Blacks, African Americans, population differences, chronic disease, chronic medical conditions, health insurance

## Abstract

Although the protective effect of health insurance on population health is well established, this effect may vary based on race/ethnicity. This study had two aims: (1) to test whether having health insurance at baseline protects individuals over a 10-year period against incident chronic medical conditions (CMC) and (2) to explore the race/ethnic variation in this effect. Midlife in the United States (MIDUS) is a national longitudinal study among 25–75 year-old American adults. The current study included 3572 Whites and 133 Blacks who were followed for 10 years from 1995 to 2004. Race, demographic characteristics (age and gender), socioeconomic status (educational attainment and personal income), and health insurance status were measured at baseline. Number of CMC was measured in 1995 and 2005. Linear regression models were used for data analysis. In the overall sample, having health insurance at baseline was inversely associated with an increase in CMC over the follow up period, net of covariates. Blacks and Whites differed in the magnitude of the effect of health insurance on CMC incidence, with a stronger protective effect for Blacks than Whites. In the U.S., health insurance protects individuals against incident CMC; however, the health return of health insurance may depend on race/ethnicity. This finding suggests that health insurance may better protect Blacks than Whites against developing more chronic diseases. Increasing Blacks’ access to health insurance may be a solution to eliminate health disparities, given they are at a relative advantage for gaining health from insurance. These findings are discussed in the context of Blacks’ diminished returns of socioeconomic resources. Future attempts should test replicability of these findings.

## 1. Background

Socioeconomic status (SES) [1], social capital [2], human capital [3], and other resources such as health insurance [4] all have health implications for populations. A wide range of SES indicators such as educational attainment [5], employment [6], and income [7] protect populations against chronic medical conditions (CMC) as well as mortality. Individuals who have access to large social networks [8], those who receive a considerable amount of social support [9], and those who are more religious [10] are more likely to stay healthy. Individuals who report high self-efficacy, mastery, and a sense of control over life also maintain better health [11,12]. Access to health insurance is essential for maintaining health status [13].

The protective effects of SES and psychosocial assets, however, are unequal across racial and ethnic groups. Blacks are at a relative disadvantage compared to Whites in gaining health from most economic resources [14,15]. Marital status [16,17], educational attainment [16,18,19,20], employment [21], and income [22,23,24,25] show stronger health effects for Whites than Blacks. These effects are shown on several health outcomes including anxiety [17], depression [23], sleep quality [26], health behaviors [18,27,28], obesity [16], oral health [25,29], self-rated health [30], CMC [22,24], and mortality [18,21]. A large social network [31] and better neighborhood quality [32] also have larger effects if the health outcomes of Whites than Blacks. The same pattern is reported for self-efficacy [33], mastery [34], and a sense of control over life [35], which all better protect the health of Whites than Blacks.

In contrast to SES and psychological assets that have larger effects for Whites than Blacks, relationships with God and others seem to have larger protective effects for Blacks in comparison to Whites. For example, social support better promotes the mental health of Blacks than Whites [36,37]. Similarly, religious involvement provides a greater mental health benefit to Blacks than Whites [36,37,38,39,40,41,42].

Although health insurance protects populations’ health [4], we are not aware of any previous studies comparing Blacks and Whites for the effect of health insurance on incidence of CMC over time. It is unclear whether the effect of health insurance status is equal or different across racial and ethnic groups. Previous literature suggests health insurance benefits show a pattern similar to economic resources, with Blacks being at a relative disadvantage compared to Whites in gaining health from it [36,37,38,39,40,41,42]. Some may expect the opposite pattern, however, as Blacks have shown larger health gains than Whites from non-economic resources such as religiosity and social support [31,32,33,34,35,36,37].

To fill the above gap in the literature, this study was conducted with two aims: (1) to test whether health insurance status at baseline predicts subsequent incidence of CMC and (2) to explore the variation in the protective effect of health insurance on incidence of CMC over time based on race/ethnicity. We did not have a specific hypothesis regarding relative advantage, or a relative disadvantage, of Blacks compared to Whites in translating health insurance access to lower incidence of CMC over time.

## 2. Methods

### 2.1. Design and Setting

Midlife in the United States (MIDUS) is a 10-year prospective cohort study that followed over 7000 American adults (aged 25–74) from 1995–2004. The main goal of the study was to discover psychosocial factors that impact aging [43,44,45,46,47,48].

### 2.2. Ethics and Institutional Review

The MIDUS study was approved by the University of Wisconsin Institutional Review Board (IRB). Participants provided a written informed consent. Monetary incentive was given to participants at both Wave 1 and Wave 2 (US $80 total).

### 2.3. MIDUS Sample Parts

The MIDUS 1 data collection is comprised of the following four components. Part 1, Main, Sibling and Twin Data include 7108 respondents. Inclusion of each of these subsections require unique set of assumptions and analytical requirements. Overall, the results are believed to be generalizable to the US sample.

### 2.4. Participants and Sampling

The MIDUS used random digit dialing (RDD), which is a sampling method for telephone surveys. RDD generates telephone numbers at random as a way to select a random sample. The MIDUS used a national RDD, selecting phone numbers from across the continental United States as the sampling frame.

### 2.5. Loss to Follow-Up and Attrition

From 7108 participants who participated in MIDUS (completed baseline phone interview), data were available for 4963 (70%) at MIDUS 2 ten years after the baseline interview. Overall, the ten-year retention rate was 75% in the MIDUS, (after adjustment for mortality as a cause for attrition). Major causes for drop out and loss to follow up were refusal, unable to contact, too ill to interview, and death [43,44,45,46,47,48].

### 2.6. Data Collection and Interviews

Data were collected using the following multiple modes: Face-to-face interview, mail questionnaire, telephone interview, computer-assisted personal interview (CAPI), and computer-assisted telephone interview (CATI) [43,44,45,46,47,48].

### 2.7. Measures and Variables

Demographic variables including gender (0 = male, 1 = female), age (continuous), and race (0 = Whites, 1 = Blacks) were collected in 1995 at baseline.

#### 2.7.1. Socioeconomic Status (SES)

Socioeconomic status was measured using education and personal income as indicators. Education attainment was measured as (1) less than high school, (2) high school graduate or equivalent, (3) some college, and (4) college graduate or more. Personal income was measured in USD. Both SES indicators were operationalized as continuous variables.

#### 2.7.2. Health Insurance Status

Presence or absence of health insurance was measured using several items related to various types of health insurance. This study treated insurance status as a dichotomous variable (any insurance 1, none 0).

#### 2.7.3. Chronic Medical Conditions (CMC)

The number of CMCs, measured at baseline and follow up, comprised of self-reported data on presence of the 12 conditions over the past year. The following conditions were assessed: high blood pressure, diabetes, stroke, asthma/chronic bronchitis/emphysema, tuberculosis, other lung disease, persistent skin problems, lower back pain, urinary/bladder problems, ulcer, bone and joint problems and hernia/rupture. Previous epidemiological studies have shown that self-reported CMC has high reliability and validity [49,50,51,52]. CMC was treated as a continuous measure.

### 2.8. Statistical Note

We used SPSS 22.0 (IBM Inc., Armonk, NY, USA) to conduct the data analyses. For univariate analysis, we used the frequency table (percentage) and mean (standard deviation; SD) in the pooled sample and by race/ethnicity. For bivariate analysis, we estimated independent sample t test and chi square test to test race/ethnic differences in study variables. For multivariable analysis, we estimated four linear regression models. Two of the models were in the pooled sample and two of the models were race/ethnic specific models. In our models, CMC at time 2 was the dependent variable, health insurance was the independent variable, and age, gender, SES, and CMC at time 1 were the covariates. In our first model, only the main effects were entered. Subsequently, we added the race/ethnicity by health insurance interaction term. Adjusted unstandardized regression coefficients (beta) with standard error (SE), 95% Confidence Intervals (CI), and *p*-values were reported.

## 3. Results

### 3.1. Descriptive Statistics

The current study included 133 Black and 3572 White adults who were followed over a 10-year period. Table 1 shows the summary of descriptive statistics for the pooled sample as well as based on race. In comparison to Whites, Blacks had a higher proportion of women. Blacks were also younger and had lower education attainment and personal income compared to Whites. Blacks had a higher number of CMC at baseline than Whites (Table 1).

### 3.2. Models in the Pooled Sample

In MIDUS, being covered by a health insurance at baseline was associated with a lower incidence of CMC over the 10 year follow up (CMC) in the pooled sample (Table 2). This protective effect was, however, significantly higher for Blacks than Whites, as evident by an interaction between race/ethnicity and baseline health insurance status on CMC incidence over time (Table 2).

### 3.3. Models in the Pooled Sample

Race/ethnic specific models also confirmed the pattern observed in the model with the interaction term. While health insurance was associated with a smaller increase in CMC over time for both race/ethnic groups, the magnitude of this effect was larger for Whites than Blacks (Table 3).

## 4. Discussion

The current study explored the race/ethnic variation in the health effects of having health insurance at baseline, in terms of CMC incidence. Insurance coverage may better protect Blacks than Whites against incidence of CMC over a 10-year period.

The minorities’ diminished return theory [14,15], shown to be valid for Blacks [16,17,18,19,20,21,22,23,24,25] and Hispanics [29], suggests that SES indicators better protect health of Whites than non-Whites. That is, promotion of marital status [16,17], education [16,18,19,20], employment [21], income [22,23,24,25], size of social network [31], quality of neighborhood [32], and coping assets [33,34,35] would result in a larger promotion of health for Whites than Blacks. Furthermore, these results are shown for mental health [17,23,26], health behaviors [18,27,28], oral health [25,29], subjective health [27], as well as physical health [16,18,21,22,24]. Specifically, SES may better protect Whites than Blacks against obesity [16], CMC [22,24], and mortality [18,21]. The additional resources attained at a higher relative SES systemically better protect Whites than Blacks against depression, chronic disease, and mortality.

At least two studies have suggested racial differences exist in the effects of expanding health insurance on health [53,54]. Such reports reduce enthusiasm for expansion of health insurances as a core strategy to eliminate the health gap by race/ethnicity [54]. The current study, however, suggests expanding health insurance as a solution to reduce the racial gap in CMC.

The minorities’ diminished returns theory, however, is more relevant to distal SES indicators such as education and occupation than proximal SES determinants such as income [14,15]. In a study, education decreased the risk of mortality for Whites than Blacks; however, this differential effect was absent for income [19]. Although not supported by all studies [22,24], some research [9] suggests that, by moving from education to income, diminished returns disappear for physical health outcomes [19]. Similarly, there are more pronounced diminished returns for mental than physical health outcomes [20,55,56,57,58]. Several studies have documented worse mental health of Backs of high SES [20,55,56,57,58].

This study, however, suggests that a universal increase in insurance coverage for Blacks and Whites may be a solution to reduce health disparities. This is an important finding because eliminating the racial gap in SES would not be enough to eliminate the racial health disparities [14,15]. This is because the same level of improvement in SES generates more health effect for the advantaged group, compared to the disadvantaged group. If the finding reported here is accurate, it is good news for health policy makers in the U.S. Similar increases in insurance coverage for Blacks and Whites may show a significant benefit for Blacks than Whites, which is desired for those who are interested in the elimination of racial and ethnic health disparities.

### 4.1. Study Limitations

This study is not free of limitations. First, data were dated. Replication of the findings is required using new data. Second, this study did not differentiate between various types of CMC. For example, insurance may have a differential protective role against development of heart disease compared to arthritis. In addition, this study did not measure mortality due to CMC, but only a crude measurement of CMC. Third, this study measured CMC using self-reported data. More research is required using other sources of data such as administrative. Fourth, several important confounders were not evaluated. Occupation, marital status, obesity, smoking, and mental health need to be included in future research. Fifth, the type of health insurance was not assessed. It is known that Blacks and Whites do not have similar types of insurance [58]. Finally, the sample size was unbalanced, as most of the MIDUS participants are White. Given the small sample of Blacks in our analysis, we did not analyze the data specific to the type of insurance. Similarly, we did not run separate models in individuals with and without CMCs at baseline. As the type of insurance is different in Whites and Blacks, and baseline CMC is more common in Blacks than Whites, the results should be replicated in the future. Although these methodological limitations were present, the current study had some strengths. Strengths included large sample size, 10 years of follow up status, and the national scope of our study. As a result, the findings still make a unique contribution to the literature by expanding the existing knowledge regarding differential effects of resources by race/ethnicity.

### 4.2. Direction for Future Research

More research is needed on this topic. First, as the data were old, and changes to insurance policies have happened in the U.S., there is a need to replicate these findings in newer data sets. Major policy changes such as Affordable Care Act (ACA) may have impacted the health gains that follow for various racial and ethnic groups [59]. Another research area that requires exploring is differential effects of various types of health insurance. This is particularly important given the Black–White differences in the types of insurance, as seen in this study. While Blacks had a higher tendency to have federal health insurance, Whites were more likely to be under private health insurance. As the health gain of being insured depends on what is covered in the health insurance plan, future research may compare Blacks and Whites with similar types of health insurance. Additionally, health insurance may have different effects depending on the health status of the insured. For example, health insurance may be particularly consequential for individuals with CMC. There is a need to compare Blacks and Whites with and without CMC (pre-existing conditions) for the health effects of health insurance [60,61]. Future research could also investigate continuity of health insurance over time. In this way, insurance status may be modeled as a time-varying covariate. Finally, there is a need to study the mechanisms by which the health effects of health insurance differ for Whites and Blacks. This is particularly important given the small sample of Blacks in this study. All issues above considered, the study findings should be regarded as preliminary.

## 5. Conclusions

In summary, in the U.S., the health return associated with health insurance may not be equal across race/ethnic groups. Future research should replicate this finding. Research may also seek reasons by which race/ethnic groups differ in how they benefit from resources such as health insurance. If these results are replicated in future attempts, increasing insurance coverage for Blacks may be a solution to the diminished returns of SES resources for them.

## Figures and Tables

**Table 1:** Descriptive statistics in all and by race.

**Variables**	**All (*n* = 3705)**		**Whites (*n* = 3572)**		**Blacks (*n* = 133)**		***P***
	***N***	**%**	***N***	**%**	***N***	**%**	
Gender							
Men	3395	47.8	2683	47.9	121	37.7	<0.001
Women	3632	51.1	2917	52.1	200	62.3	
Health Insurance							
No	239	6.5	214	6.0	25	18.8	<0.001
Yes	3466	93.5%	3358	94.0	108	81.2	
**Variables**	**Mean**	**SD**	**Mean**	**SD**	**Mean**	**SD**	***P***
Age	46.38	13	47.3	12.92	44.42	12.54	<0.001
Education	6.77	2.49	6.9	2.47	6.22	2.47	<0.001
Income	26,773.24	26,891.19	27,326.10	27,509.71	20,762.54	19,730.37	<0.001
Chronic Medical Conditions	2.41	2.51	2.39	2.46	2.53	2.96	0.422

**Table 2:** Linear regression in the pooled sample based on the association between healthcare insurance and incidence of chronic medical conditions (CMC) over 10 years.

Variables	Model 1 (All)				Model 2 (All)			
	Beta	SE	95% CI	*p*	Beta	SE	95% CI	*p*
Race (Black)	0.81	0.19	(0.44, 1.18)	<0.001	2.38	0.45	(1.49, 3.27)	<0.001
Age	0.03	0.00	(0.02, 0.03)	<0.001	0.03	0.00	(0.02, 0.03)	<0.001
Gender (Female)	0.22	0.08	(0.07, 0.37)	0.004	0.21	0.08	(0.07, 0.36)	0.005
Education (Years)	−0.06	0.02	(−0.09, −0.03)	<0.001	−0.06	0.02	(−0.09, −0.03)	<0.001
Income (Personal)	−0.01	0.00	(0.00, 0.00)	0.510	−0.01	0.00	(0.00, 0.00)	0.496
CMC time 1	0.53	0.02	(0.50, 0.56)	<0.001	0.53	0.02	(0.50, 0.56)	<0.001
Health Insurance	−0.50	0.14	(−0.78, −0.22)	0.001	−0.33	0.15	(−0.63, −0.04)	0.028
Race (Black) × Health Insurance	-	-	-	-	−1.90	0.50	(−2.87, −0.92)	<0.001
Constant	0.51	0.25	(0.02, 1.00)	0.042	0.37	0.25	(−0.13, 0.86)	0.144

Dependent Variable: Chronic Medical Conditions (CMC) time 2.

**Table 3:** Linear regressions in Whites and Blacks based on the association between healthcare insurance and incidence of chronic medical conditions (CMC) over 10 years.

Variables	Model 3 (Whites)				Model 4 (Blacks)			
	Beta	SE	95% CI	*p*	Beta	SE	95% CI	*p*
Age	0.02	0.00	(0.02, 0.03)	<0.001	0.04	0.04	(−0.03, 0.11)	0.227
Gender (Female)	0.20	0.07	(0.06, 0.34)	0.006	0.40	0.92	(−1.43, 2.22)	0.667
Education (Years)	−0.04	0.01	(−0.07, −0.01)	0.009	−0.46	0.16	(−0.79, −0.14)	0.006
Income (Personal)	−0.02	0.00	(0.00, 0.00)	0.070	0.00	0.00	(0.00, 0.00)	0.024
CMC time 1	0.54	0.01	(0.51, 0.56)	<0.001	0.44	0.20	(0.05, 0.83)	0.029
Health Insurance	−0.33	0.14	(−0.61, −0.06)	0.016	−2.44	1.07	(−4.57, −0.32)	0.025
Constant	0.34	0.24	(−0.12, 0.81)	0.146	3.61	2.83	(−2.00, 9.21)	0.205

Dependent Variable: Chronic Medical Conditions (CMC) time 2.
